# The potential role of microvascular pathology in the neurological manifestations of coronavirus infection

**DOI:** 10.1186/s12987-020-00216-1

**Published:** 2020-09-10

**Authors:** M. A. MacLean, L. Kamintsky, E. D. Leck, A. Friedman

**Affiliations:** 1 Division of Neurosurgery, Dalhousie University, Queen Elizabeth II Health Sciences Centre (Halifax Infirmary), 1796 Summer Street, Halifax, NS B3H 3A7 Canada; 2 Department of Medical Neuroscience, Faculty of Medicine, Dalhousie University, Room 12 H, 12th Floor, Sir Charles Tupper Building, 5850 College Street, PO Box 15000, Halifax, NS Canada

**Keywords:** Blood–brain barrier, Neurological, Coronavirus, SARS-CoV-2, Neurology, Stroke

## Abstract

Human coronaviruses are highly pathogenic viruses that pose a serious threat to human health. Examples include the severe acute respiratory syndrome outbreak of 2003 (SARS-CoV-1), the Middle East Respiratory Syndrome (MERS-CoV) outbreak of 2012, and the current SARS-CoV-2 (COVID-19) pandemic. Herein, we review the neurological manifestations of coronaviruses and discuss the potential pathogenic role of blood–brain barrier dysfunction. We present the hypothesis that pre-existing vascular damage (due to aging, cardiovascular disease, diabetes, hypertension or other conditions) facilitates infiltration of the virus into the central nervous system (CNS), increasing neuro-inflammation and the likelihood of neurological symptoms. We also discuss the role of a neuroinflammatory cytokine profile in both blood–brain barrier dysfunction and macrovascular disease (e.g. ischemic stroke and thromboembolism). Future studies are needed to better understand the involvement of the microvasculature in coronavirus neuropathology, and to test the diagnostic potential of minimally-invasive screening tools (e.g. serum biomarkers, fluorescein retinal angiography and dynamic-contrast MRI).

## Introduction

Coronaviruses are enveloped viruses found in animals and humans. Coronaviruses possess a single-stranded RNA genome that encodes structural proteins that allow binding to host cells, and replicase proteins that allow viral replication [1]. The first coronavirus causing severe acute respiratory syndrome (SARS-CoV-1) was identified as a clinical entity in 2002 [2]. This was followed by the emergence of the Middle East Respiratory Distress Syndrome (MERS-CoV) in 2012 [3], and SARS-CoV-2 in 2019 [4, 5].

Severe cases of coronavirus infection are associated with acute microvascular disease of the respiratory system [6]. Clinically, this manifests as failure to breath due to widespread lung inflammation, termed acute respiratory distress syndrome (ARDS) [7–9]. Risk factors for ARDS are old age and comorbidities such as cerebrovascular disease, diabetes, and hypertension [10, 11]. These conditions are associated with immune and vascular dysfunction, and predispose patients to severe infection [10–16].

In addition to the established role of coronavirus infection in respiratory system dysfunction, accumulating evidence associates coronaviruses with neuropathology [6, 8, 9, 17–24]. Herein, we review the neurological manifestations of coronavirus infection and discuss the potential contribution of microvascular pathology and both systemic and central nervous system (CNS) inflammation. We present the hypothesis that pre-existing endothelial disease (due to ageing, cardiovascular disease, diabetes, hypertension and other conditions) may facilitate infiltration of the virus into the CNS, increasing neuroinflammation and the likelihood of neuropathology.

### Neurological manifestations of coronaviruses

A recent cohort study from Wuhan, China reported neurological findings in 24.8% of 214 adult patients with SARS-CoV-2 [19]. Neurological findings were associated with severe infection, older age, and multiple comorbidities [10]. Findings included encephalitis, epileptic seizures, ischemic and hemorrhagic stroke, delirium, headache, dizziness, impaired consciousness, and ataxia. Recent reviews highlight the significant incidence of neurological symptoms following SARS-CoV-2 infection [21–26], including gustatory and olfactory dysfunction (e.g. anosmia), occurring early in the course of disease following SARS-CoV-2 infection [23, 27–29].

Human coronavirus infection was also found to cause neurological symptoms in children [30]. While children under the age of 10 represent less than 1% of cases [31] and have a milder course of illness compared to adult patients [32], emerging case series describe severe neurological symptoms among children as well [30]. Symptoms are similar to those seen in infected adult patients [19], and may involve the central and peripheral nervous systems [30].

Neurological symptoms were also reported in SARS-CoV-1 and MERS-CoV patients, and included confusion, coma, ataxia, stroke, and focal motor deficits [8, 9]. Magnetic Resonance Imaging (MRI) in a select group of MERS-CoV patients revealed widespread bilateral lesions within the white matter, subcortical areas, basal ganglia, corpus callosum, pons, cerebellum and upper cervical spinal cord [9]. Autopsy studies of patients who died of SARS-CoV-1 confirmed the presence of the virus in the brain [33]. Solomon et al. recently described the post-mortem neuropathological changes associated with SARS-CoV-2 infection in 18 patients. They detected virus in low levels within the brain of several patients with pre-existing microvascular disease (e.g. diabetes, hypertension, cardiovascular disease), all of whom presented with encephalopathy [34]. Their work highlights the importance of pre-existing comorbidity in predisposing to acute hypoxic ischemic brain injury.

## Potential routs of CNS invasion

**Fig. 1:**
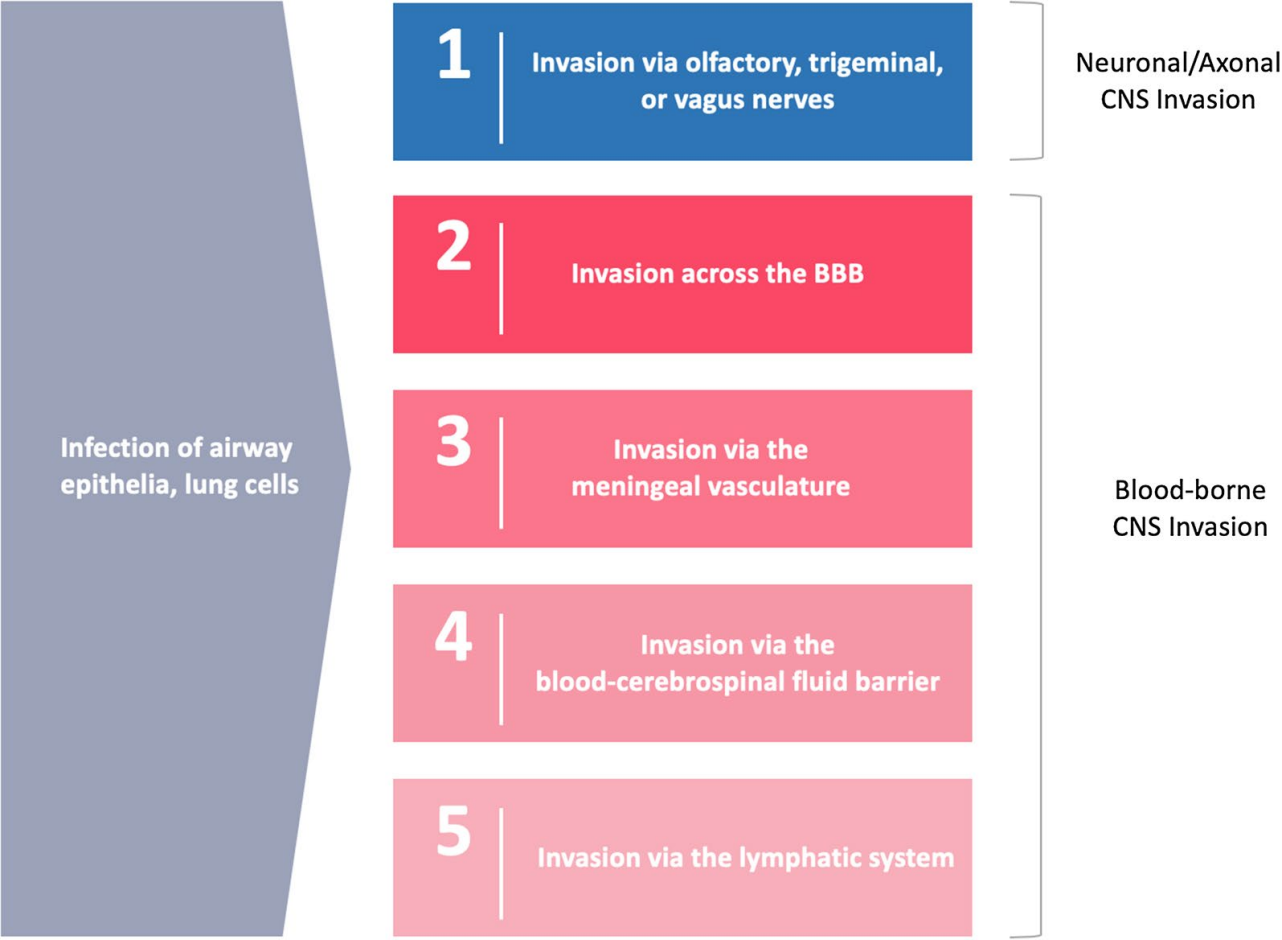
Potential routes of coronavirus CNS neuroinvasion

Human-to-human transmission of coronaviruses is primarily mediated by respiratory droplets [35]. Coronaviruses utilize spike proteins to bind cell receptors: the hDPP4 receptor serves as the binding site for MERS-CoV, while the ACE2 receptor allows the binding of SARS-CoV-1 and SARS-CoV-2 [1, 3]. These receptors are expressed in human airway epithelia, lung parenchyma and nerve tissue [36, 37]. Both receptors are also expressed in vascular endothelia, including the endothelial cells of the cerebral microvasculature [3, 18, 33, 37–39]. Recently, it has been shown for the first time that SARS-CoV-2 directly infects endothelial cells in vivo [40]. Human coronaviruses demonstrate tropism for neuronal cells, yet the routes by which they may reach the brain remain only partially understood. Two leading hypotheses suggest that coronaviruses can reach the CNS through neuronal-axonal transport or through the bloodstream [33] (Fig. 1).

1. Neuronal-axonal CNS invasion
  Infection of the olfactory nerve may allow invasion of the CNS, resulting in widespread expression of respiratory viruses in the brain [18, 33, 41, 42]. Neuronal-axonal neuroinvasion of coronaviruses has been demonstrated in animals following intranasal inoculation [33]. Neuroinvasion of olfactory sensory neurons may partly explain the pathogenic mechanism of gustatory and taste dysfunction associated with SARS-CoV-2 infection. Another plausible mechanism involves damage secondary to direct viral binding to ACE2 receptors on the olfactory epithelium [27, 28].
  Although olfactory sensory neurons in the neuroepithelium of the nasal passage are vulnerable to neuroinvasion, respiratory infections rarely cause fatal encephalitis [43]. Moseman et al. demonstrated that brain-resident microglia provide innate defense against neuroinvasive nasal infection (by cross-presenting antigens to anti-viral T cells after acquisition from adjacent neurons, and non-cytolytic cleansing of neurons) [43]. To this end, microglial dysfunction function may predispose to neuroinvasion. SARS-CoV-1 infected neurons produce proinflammatory cytokines (e.g. IL-1b, TNF-alpha, IL-6) which may be neurotoxic [18].
  The vagus nerve is another potential pathway for neuroinvasion, as it was previously implicated in the spread of influenza from the respiratory tract to the brainstem [44]. Trans-vagal transport has also been reported for neurotropic enteroviruses, reoviruses, and others [44]. Autonomic axons may be more vulnerable to neuroinvasion by viruses due to reduced myelination [44].
2. CNS invasion across the BBB
  The microvasculature of the brain is comprised of several components: endothelial cells connected with tight junction proteins, pericytes that wrap around the endothelial cells, and astrocytes—whose end-feet cover most of the surface area of the vasculature. Together with nearby neurons, these cells form the neurovascular unit, the building blocks of the BBB (Fig. 2a) [45]. SARS-CoV-1 and MERS-CoV were shown to directly bind endothelial cells and smooth muscle in the cerebral microvasculature which express ACE2 and DPP4 receptors, respectively [3, 33, 38, 46–48]. As such, SARS-CoV-2 may also be capable of directly binding to the endothelial cells of the BBB, leading to direct neuroinvasion, as documented with SARS-CoV-1 infection [33, 38].

**Fig. 2:**
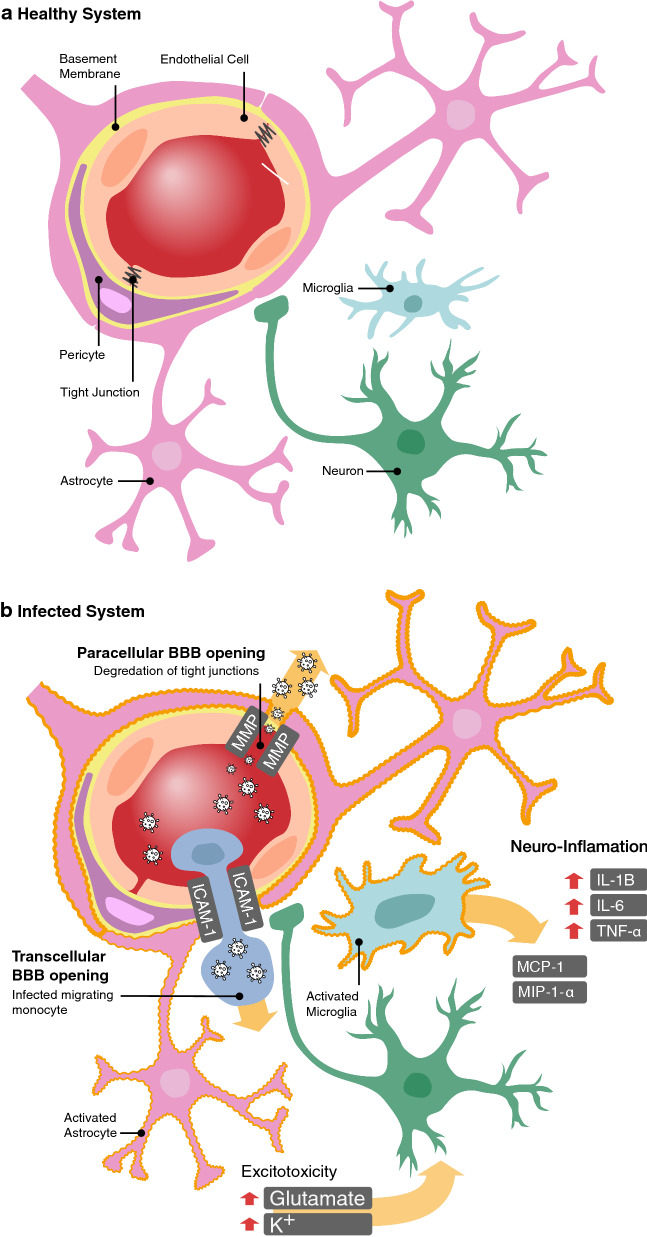
The neurovascular unit in health and coronavirus infection. **a** Under normal conditions the brain’s microvasculature (i.e. the blood–brain barrier, BBB) restricts the entry of most macromolecules and neurotoxins in the bloodstream from entering the brain. This protection of the neuronal tissue is achieved by the components of the neurovascular unit: endothelial cells (tightly connected by tight junction proteins), pericytes (wrapped around the endothelium), astrocytes (whose end-feet cover most of the surface area of the vasculature), and the nearby microglial cells. **b** Accumulating evidence suggests that coronaviruses are able to invade the brain. Here we depict two potential routs of invasion across the BBB. Coronaviruses may enter the bloodstream from the airway, and directly infect the endothelial cell of the BBB or infect monocytes that later migrate across the BBB (through ICAM-1 facilitated transcellular transport). The presence of the virus in the brain tissue will trigger an inflammatory cascade, that involves astrocyte and microglia activation, and secretion of pro-inflammatory cytokines (IL-1β, IL-6, TNF-α) and chemokines (MPC-1, MIP-1-α). At a later stage TNF-α may upregulate the release of MMPs—enzymes that degrade the tight junction proteins and allow further paracellular leakage across the BBB. Astrocytes may also undergo a transformation to express less glutamate and potassium receptors, leading to reduced glutamate and potassium clearance, and subsequent hyper-excitability and seizures
  Coronaviruses may also gain access to the bloodstream via the airway and infect circulating immune cells (e.g. monocytes) [1, 33]. Infected monocytes may cross the BBB, a process facilitated by proinflammatory cytokines and chemokines [49]. Passage of infected monocytes across the BBB may occur via a transcellular pathway involving ICAM-1-mediated transport (Fig. 2b), which is upregulated by the pro-inflammatory cytokine TNF-alpha.
  Alternatively, paracellular leakage may occur due to increased MMP-9 enzyme activity (upregulated by TNF-alpha) and subsequent degradation of tight junction and the basement membrane (Fig. 2b) [45]. Monocytes, T cells, and dendritic cells all express and release MMP-9 [45]. In addition to compromising BBB integrity, MMPs activate neuroinflammatory pathways and provide signals for further production and secretion of pro-inflammatory cytokines [45]. Following neuroinvasion, infected macrophages or microglia produce chemokines recruiting more infected leukocytes, and pro-inflammatory cytokines (e.g. TNF-alpha) that may be neurotoxic [33].
3. CNS Invasion via the blood-cerebrospinal fluid (B-CSF) barrier
  Many viruses invade the CNS via infection of epithelial cells of the blood-cerebrospinal fluid (B-CSF) barrier in the choroid plexus, located in the ventricles of the brain [50, 51]. The choroid plexus is an epithelia-endothelial convolute with highly vascularized stroma, fenestrated capillaries, and epithelial cells joined by tight junctions [51]. Systemic inflammation following exposure to bacterial endotoxins, blood products following hemorrhage, and injury following ischemic stroke, alters gene expression in the choroid plexus. Affected genes can in turn regulate the immune response, extra-cellular matrix remodelling, and B-CSF barrier integrity [52]. Such processes may activate NF-kB, resulting in upregulated MMP-9 production, and altered B-CSF barrier permeability [53]. These processes were also shown to upregulate other factors that affect B-CSF barrier permeability and immune cell trafficking (e.g. MMP-8, TNF-alpha, IL-6, IL-1B, MCP-1, and ICAM-1) [51, 54–56]. The role of the choroid plexus and circumventricular organs in SARS-CoV-2 neuropathology remains unknown. However, ACE2 is expressed in circumventricular organs such as the subfornical organ and organum vasculosum of the lamina terminalis [36]. The epithelial-endothelial convolute of the choroid plexus may provide a reservoir for SARS-CoV-2 and facilitate entry across the B-CSF barrier [51].
4. CNS Invasion via the meningeal vasculature
  The meninges may represent another site of trafficking of infected immune cells into the CNS [55]. Adhesion molecules expressed in the blood–brain and blood-CSF barrier of the choroid plexus (e.g. ICAM-1, P-selectin, E-cadherin), are also expressed in the meningeal vasculature. It is unclear if meningeal tissue becomes infected with SARS-CoV-2. Clinically, meningitis is observed following infection with various respiratory viruses, including human coronaviruses [19, 25, 57]. Solomon et al. has recently reported post-mortem evidence of focal leptomeningeal inflammation in a single patient [34].
5. CNS Invasion via the lymphatic system
  In autopsy samples, SARS-CoV-1 viral particles have been detected in lymphocytes, lymph nodes, and lymphatic organs—findings that are in line with the lymphopenia observed following infection [6]. Lymphopenia is also observed with SARS-CoV-2 infection [11]. However, the role of the lymphatic system in predisposing patients to neuroinvasion in unclear.

## BBB dysfunction, systemic and CNS inflammation, and viral neuroinvasion

ACE2 plays a role in attenuating microvascular pathology [58], conferring protection against capillary endothelial dysfunction, atherogenesis, thrombus formation, oxidative stress, and inflammatory cascades responsible for monocyte-endothelial cell interactions [48, 59]. Given that SARS-CoV-2 has recently been shown to directly bind ACE2 on endothelial cells in vivo [40], microvascular dysfunction may occur following direct viral binding of ACE2 expressed on the capillary endothelium of the BBB, triggering an inflammatory cascade [47, 48, 59, 60]. Many proinflammatory cytokines (e.g. IL-6, IL-3, TNF-alpha) have neurotoxic potential [61]. Furthermore, proinflammatory cytokines and chemokines (e.g. MIP-2, IL-8, IL-6, MCP-1) increase BBB permeability and facilitate monocyte and leukocyte transmigration across the BBB, propagating damaging neuroinflammatory pathways (Fig. 2b) [49, 62]. As this effect is diminished in animals without functioning immune cells, they are thought to be directly involved in neuroinvasion across the BBB [49].

Adult patients with coronavirus infection develop a systemic pro-inflammatory cytokine response (i.e. increased levels of IL-1B, TNF-alpha, VEGF, IL-2, IL-6, IL-7, IL-12, granulocyte-colony stimulating factor, IFN-gamma, IP-10, MCP-1, and MIP-1-alpha) [7, 63–67], which likely contributes to BBB dysfunction [45]. Levels of cytokines and chemokines (i.e. MCP-1, TGF-beta, IP-10, IL-6, IL-8, GM-colony stimulating factor) are also elevated in the CSF of adult [68, 69] and pediatric patients with neurological symptoms and coronavirus infection [70]. Following SARS-CoV-2 infection, neuroinflammatory cytokines (e.g. TNF-alpha) may also upregulate MMP-9 activity, allowing leakage of neurotoxic factors or infected immune cell migration across the BBB [33, 45].

**Fig. 3:**
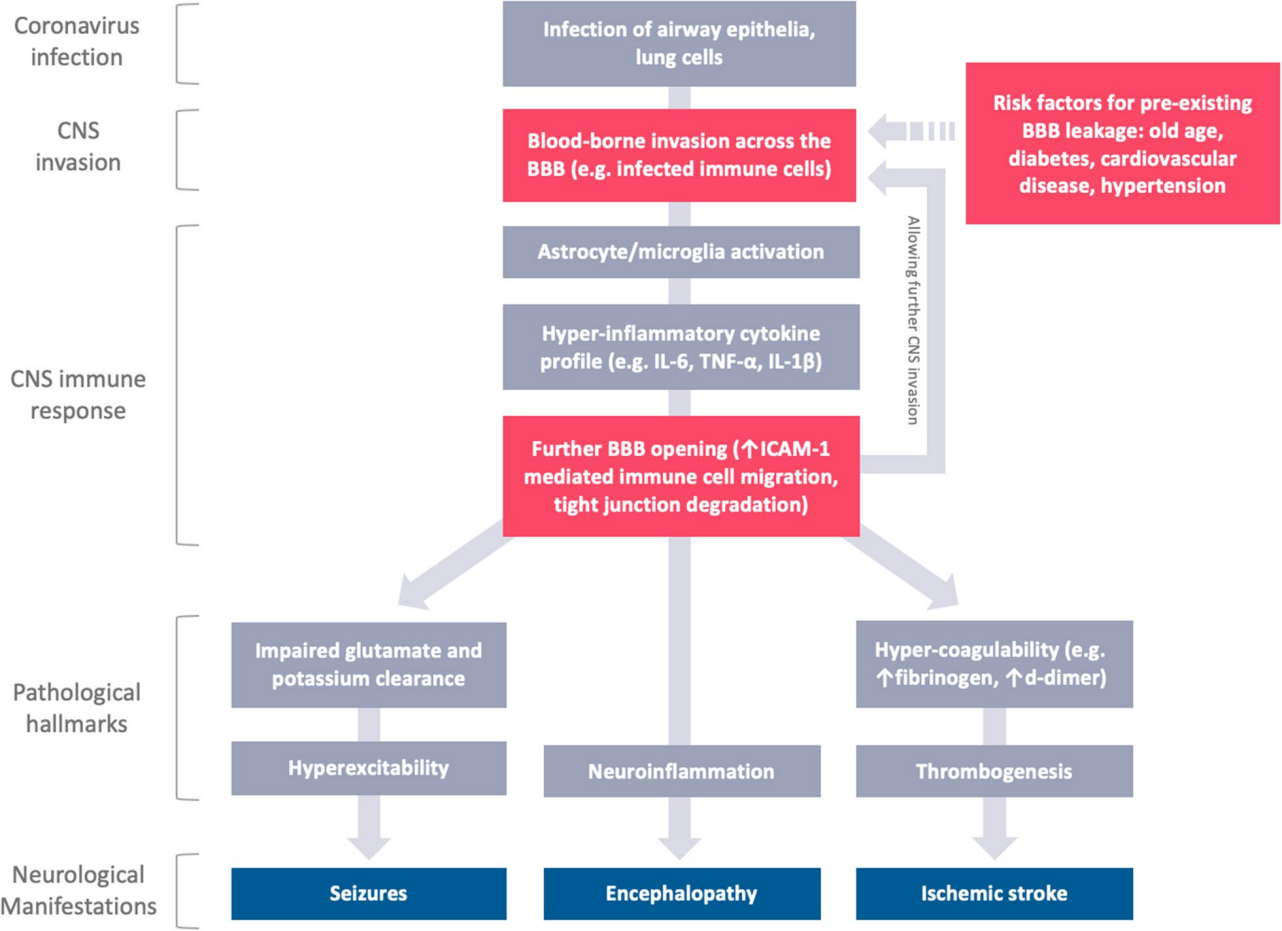
The proposed pathophysiology of the neurological manifestations of coronavirus infection. *BBB* blood–brain barrier

We hypothesize that patients with pre-existing BBB dysfunction are likely to be at higher risk of CNS invasion of coronaviruses (Fig. 3). Notably, microvascular damage, and specifically dysfunction of the BBB, are common features of aging and the diseases most-associated with coronavirus infection severity and neurological symptoms (e.g. cerebrovascular disease, diabetes mellitus, and hypertension) [10–16, 34]. Together, these findings suggest that damage to the BBB, either due to a pre-existing comorbidity or virus-induced pro-inflammatory response, may facilitate the extravasation of infected immune cells from the bloodstream to the brain.

## Effects of coronaviruses on neuronal tissue

Once the virus has reached neuronal tissue, it can trigger a neuroinflammatory response leading to hyper-excitability, neurodegeneration and death [33].

### Coronavirus-related ischemic and hemorrhagic stroke

ACE2 has been shown to attenuate inflammation, thrombus formation and platelet aggregation [58, 71]; given that SARS-CoV-2 directly binds ACE2 in blood vessels, this is a likely source of microvascular dysfunction [40, 72, 73]. Loss of ability to prevent thrombosis likely occurs following cell entry of SARS-CoV-2 [72]. Several studies have associated SARS-CoV-2 infection with stroke and vasculopathy in both young and old patients [23, 24, 74–79]. The likelihood of ischemic stroke is eight times higher in patients with SARS-CoV-2, compared to influenza [80]. SARS-CoV-2 is associated with a prothrombotic state causing arterial and venous thromboembolism [81]. Patients have markedly elevated D-dimer, and consumption of fibrinogen. Abnormal coagulation markers have been associated with poor prognosis among patients with SARS-CoV-2 infection [81]. The exact mechanisms underpinning increased stroke rates remain to be further elucidated.

Following ischemic stroke, BBB function is further augmented, due to multiple mechanisms, including neuronal depolarization (i.e. spreading depolarization and/or seizures), high levels of glutamate, and increased levels of MMPs [45, 82]. This results in neuroinflammation, brain parenchymal damage, and other complications (e.g. seizures, and hemorrhagic transformation) [45, 82].

### Coronavirus-related encephalopathy and meningitis

Adults infected with SARS-CoV-2 may develop acute encephalopathy and meningitis [21–24, 83], with neuroimaging evidence of microhemorrhages and diffuse inflammation of the white matter [79]. A case of SARS-CoV-2 infection was associated with acute hemorrhagic necrotizing encephalopathy (ANE) [84]. Encephalopathy has also been described among children infected with SARS-CoV-2 [30, 85]. Brain MRI changes in the splenium of the corpus callosum of pediatric patients infected with SARS-CoV-2 [30], are similar to those reported in children with Kawasaki disease [86]. Li et al. examined the CSF of 183 children hospitalized with encephalitis-like syndrome following coronavirus infection and found elevated CSF levels of IL-6, IL-8, and MCP-1 [30]. A recent case series in adult patients with SARS-CoV-2 infection-related encephalopathy confirmed these findings [68].

Among patients with viral encephalitis and seizures, viral RNA is not always identified in the CSF [83]. Rather, CSF antibody levels (e.g. IgM) may indicate neuroinvasion, as observed with other viral encephalitides [68]. SARS-CoV-2 is detected in the blood of patients with active infection in only 1% of cases, indicating failure to detect the virus in CSF does not decrease the likelihood of direct CNS neuroinvasion [87]. Although detection of SARS-CoV-2 in the CSF of infected patients presenting with meningoencephalitis has been reported [88, 89], a case series provides evidence in support of SARS-CoV-2 direct CNS neuroinvasion in the absence of viral RNA in the CSF [68]. Rather, antibodies to spike and envelope proteins were identified.

Evidence of SARS-CoV-1-induced neuroinflammation was previously demonstrated post-mortem [19]. In a case of fatal encephalitis secondary to SARS-CoV-1 clinical and post-mortem examination documented cerebral edema, hemodynamic dysfunction, and brainstem herniation and necrotic neurons infected by SARS-CoV-1 [17]. Studies in mice transgenic for the receptor for MERS-CoV or SARS-CoV-1, confirmed that the viruses is found to mainly affect the thalamus and brainstem [3, 41, 42, 44]. However, ANE may also be due to intracranial cytokine storms, associated with BBB breakdown, without direct viral invasion [33, 84].

### Coronavirus-related seizures

Epileptic seizures were also reported in patients with SARS-CoV-2 [10, 23], and Galanopoulou et al. have recently identified epileptiform discharges in ~ 41% of SARS-CoV-2 patients under EEG investigation for acute encephalopathy and/or seizure‐like events [83]. BBB dysfunction and the neuroinflammatory response (e.g. IL-1-beta, IL-6 and TNF-alpha) [33] may contribute to the development of epileptiform activity [33, 66, 82, 90–93], by affecting conductance through NMDA receptors, altering astrocytic functions, as well as glutamate and potassium homeostasis [33, 65, 66].

Following seizures, adult patients with epilepsy were found to have increased levels of CSF MMPs, that were associated with post-seizure BBB leakage (Fig. 2b) [45]. MMP-9 may also mediate seizure-induced neuronal cell death by stimulating glutamate receptor-mediated excitotoxicity [45].

### A Proposed mechanism for brainstem-mediated respiratory failure

Approximately 55% of ICU admitted patients develop ARDS, and two studies reported that SARS-CoV-2 infected patients in an ICU were incapable of spontaneous breathing [7, 78]. Since spontaneous breathing is controlled by the brainstem, infection of the CNS has been postulated to contribute to respiratory dysfunction in a subset of patients [94]. This theory is supported by animal studies showing corona and influenza viruses within the brainstem of mice but has not been confirmed for SARS-CoV-2 [3, 18, 44]. Furthermore, ACE2 is expressed in key brain regions involved in the regulation of cardiovascular and respiratory function, including: subfornical organ, magnocellular neurons of the paraventricular nucleus, the area postrema, the dorsal motor nucleus of the vagus, the nucleus of the tractus solitarii, the nucleus ambiguus, and rostral ventrolateral medulla [36].

## Future directions

### Can screening for BBB dysfunction serve as a novel method for identifying high-risk patients?

Screening infected patients for BBB injury may allow early detection of patients at risk for severe outcomes. The current gold standard for measuring BBB integrity is dynamic contrast-enhanced MRI [14, 95–97]. However, the costly and time-intensive nature of the scan poses a difficulty in screening large volumes of patients. The assessment of the eye’s microvasculature using fluorescein retinal angiography may provide a cost- and time- effective alternative [98, 99]. This technique has been used to identify microvascular pathology in critically-ill ICU patients [98], and has detected increased retinal permeability in a SARS-CoV-1 patient prior to the development of severe CNS symptoms (progressive cerebral edema, hemorrhage, and brainstem dysfunction) and ARDS [17]. Serum biomarkers for BBB integrity are also under development, mainly for traumatic brain injury, with the goal of detecting brain proteins (e.g. ubiquitin C-terminal hydrolase-L1 (UCH-L1), glial fibrillary acidic protein (GFAP), neurofilament, tau, neuron-specific enolase) within the serum [100]. As the first serum biomarkers (UCH-L1 and GFAP) were recently approved by the FDA, this approach may be proven as a fast screening tool for patients at high-risk of severe infection outcomes prior to the development of neurological symptoms.

Future studies are needed to confirm the role of the BBB in coronavirus pathology. Studies should (a) investigate the association between vascular dysfunction, neurological symptoms, and ARDS in infected individuals; and (b) examine the potential of serum and imaging biomarkers for early identification of infection outcome.

## Summary

Herein, we reviewed literature that supports the role of microvascular pathology in the neurological manifestations of coronavirus infection. Integrity of the cerebral microvasculature may play an important role in regulating viral neuroinvasion. However, neuroinvasive mechanisms require further study. We propose that pre-existing microvascular pathology (due to aging, cardiovascular disease, diabetes or hypertension) contributes to the development of the CNS-related symptoms and pathology associated with coronavirus infection. Future studies are needed to better understand the involvement of the microvasculature in coronavirus pathology, and to test the diagnostic potential of minimally-invasive screening tools (e.g. serum biomarkers, fluorescein retinal angiography and dynamic-contrast MRI).

## Data Availability

Not applicable.
